# Deep-learning based quantification model for hip bone marrow edema and synovitis in patients with spondyloarthritis based on magnetic resonance images

**DOI:** 10.3389/fphys.2023.1132214

**Published:** 2023-03-03

**Authors:** Yan Zheng, Chao Bai, Kui Zhang, Qing Han, Qingbiao Guan, Ying Liu, Zhaohui Zheng, Yong Xia, Ping Zhu

**Affiliations:** ^1^ Department of Clinical Immunology, Xijing Hospital, Fourth Military Medical University, Xi’an, China; ^2^ National Translational Science Center for Molecular Medicine, Xi’an, China; ^3^ National Engineering Laboratory for Integrated Aero-Space-Ground-Ocean Big Data Application Technology, School of Computer Science and Engineering, Northwestern Polytechnical University, Xi’an, China; ^4^ Department of Radiology, Xijing Hospital, Fourth Military Medical University, Xi’an, China

**Keywords:** spondyloarthritis, hip, magnetic resonance imaging, synovitis, deep learning

## Abstract

**Objectives:** Hip inflammation is one of the most common complications in patients with spondyloarthritis (SpA). Herein, we employed use of a deep learning-based magnetic resonance imaging (MRI) evaluation model to identify irregular and multiple inflammatory lesions of the hip.

**Methods:** All of the SpA patients were enrolled at the Xijing Hospital. The erythrocyte sediment rate (ESR), C-reactive protein (CRP), hip function Harris score, and disease activity were evaluated by clinicians. Manual MRI annotations including bone marrow edema (BME) and effusion/synovitis, and a hip MRI scoring system (HIMRISS) assessment was performed by experienced musculoskeletal radiologists. The segmentation accuracies of four deep learning models, including U-Net, UNet++, Attention-Unet, and HRNet, were compared using five-fold cross-validation. The clinical agreement of U-Net was evaluated with clinical symptoms and HIMRISS results.

**Results:** A total of 1945 MRI slices of STIR/T2WI sequences were obtained from 195 SpA patients with hip involvement. After the five-fold cross-validation, U-Net achieved an average segmentation accuracy of 88.48% for the femoral head and 69.36% for inflammatory lesions, which are higher than those obtained by the other three models. The UNet-score, which was calculated based on the same MRI slices as HIMRISS, was significantly correlated with the HIMRISS scores and disease activity indexes (*p* values <0.05).

**Conclusion:** This deep-learning based automatic MRI evaluation model could achieve similar quantification performance as an expert radiologist, and it has the potential to improve the accuracy and efficiency of clinical diagnosis for SpA patients with hip involvement.

## Highlights

1. MRI is sensitive to early diagnosis of hip inflammation in SpA. A deep learning-based MRI evaluation model for hip inflammation could overcome empirical bias, improve operation efficiency, and promote aggressive treatment.
2. The UNet-score could quantify bone marrow edema and synovitis/effusion in the hip joint beyond the objects type of previous model. It could achieve quantification performance similar to that of an expert radiologist, and provide an important basis for accurately assessing the disease activity of spondyloarthritis.
3. The automatic MRI evaluation model is beneficial to optimize the comprehensive management and improve the prognosis of SpA.

## Introduction

Hip involvement is a common complication in approximately 10%–50% of spondyloarthritis (SpA) patients (Guan et al., 2013; Wink et al., 2019). Progressive hip involvement has been reported to be associated with more severe spinal involvement and disability, leading to poor prognostics (Bethi et al., 2013; Vander et al., 2013). In addition, the principal clinical manifestation of hip involvement in SpA patients suffers from intractable pain, due mainly to subchondral bone marrow edema (BME) and synovitis effusion (Appel et al., 2006). Early diagnosis and treatment could significantly prevent SpA progression, protect joint function, and avoid the development of disabilities and late-stage irreversible damage requiring joint replacement.

Magnetic resonance imaging (MRI) has been widely used and proven to be a sensitive tool for detecting early inflammation before structural damage (Huang et al., 2016; Krabbe et al., 2020). Quantitative evaluation of MRI lesions of hip inflammation may facilitate early detection and precise assessment of disease progression (Zhang et al., 2021). The hip MRI inflammation scoring system (HIMRISS) is a quantitative assessment method used to systematically assess synovitis and BME of the femoral head and acetabular (Maksymowych et al., 2014). However, traditional MRI analyses rely on the experience of a radiologist, while the concordance rate among specialists remains around 0.68–0.73 (Lopez-Medina et al., 2020). Therefore, the traditional time-consuming and experience-dependent MRI diagnosis of hip inflammation may be inadequate for long-term management and comprehensive evaluation of treatment effects.

Deep learning-based MRI evaluation models have been attempted to overcome empirical bias and errors by significantly improving the reliability and efficiency of MRI diagnosis (Hallinan et al., 2021; Zheng et al., 2022). Deep learning models have several advantages, including more objective and fast process, reduced cost of training high-level clinical specialists, and provision of a platform for large-scale screening (Lin et al., 2022). The focus of early computer-assisted image processing technology was on facilitating readings by clinical specialists *via* enhancing the medical images in which subtle data differences could be distinguished. This was achieved by processing the values of pixels in MR images or automatically segmenting boundaries and objects using active shape models on video fluoroscopic images (Jin et al., 2021; Wang et al., 2021). For deep learning, a branch of machine learning, features of layers can be studied automatically, enabling the detection of an abnormality from medical images (Zheng et al., 2022).

In preliminary work, we developed a 3D-UNet algorithm for automatic grading of BME in hip arthritis of ankylosing spondylitis (AS) patients using MRI (Han et al., 2021a). On the basis of previous work and to expedite the diagnostic process to improve the accuracy of diagnosing hip joint BME and synovitis, this study proposes a fully automatic and visualized diagnostic system based on U-Net for SpA patients with hip involvement. The accuracy of lesion detection and diagnosis was evaluated by experienced radiologists and rheumatologists. In addition, the clinical interpretability and clinical relevance of this model was further verified by comparison with current HIMRISS system and disease activity indexes.

## Materials and methods

### Study design and ethics statements

This single-center retrospective study was conducted in accordance with the ethical standards of Xijing Hospital of China. The ethics committee of Xijing Hospital of China approved this study (Instruction number: 20110303-7). Written informed consent was not required for this study due to its retrospective diagnostic study design.

### Patients and experimental setting

All of the patients enrolled in our study had a clinical diagnosis of axial SpA(axSpA). Their long-term treatment and care were initiated by rheumatologists at Xijing Hospital. Eligible patients were 18–65 years of age and had to fulfill the recently published Assessment of Spondyloarthritis International Society (ASAS) classification criteria (van der Linden et al., 1984; Zeidler and Amor, 2011) or the Modified New York criteria for axial spondyloarthritis (Deodhar et al., 2014). Both acute and chronic inflammatory changes on MRI were considered positive signs for hip involvement and coxitis. Participants were randomly divided into a training dataset and a testing dataset by 5-fold cross-validation. Among them, 110 had bilateral hip involvement and 85 had unilateral hip involvement (Table 1). The normal hips of patients were used as the control group. A total of 195 patients (a total of 390 hips) were involved in this study.

**TABLE 1:** Demographic, clinical, laboratory, and MRI characteristics of the study population. VAS, visual analogue scale; ESR, erythrocyte sediment rate; CRP, C-reactive protein; BASDAI, Bath Ankylosing Spondylitis Disease Activity Index; ASDAS, Ankylosing Spondylitis Disease Activity Score; DMARD: Disease-modifying anti-rheumatic drug; MTX, sulfasalazine; SSZ, Sulfasalazine.

	Study population (*n* = 195)
Age (years)	28.63 ± 12.04
Male	79.2%
B27	96%
Disease duration (months)	6.48 ± 5.28
VAS	3.68 ± 2.06
ESR (mm/h)	18.25 ± 14.76
CRP (mg/dL)	1.18 ± 0.76
Harris	80.68 ± 14.10
BASDAI	4 ± 2.11
Morning stiffness (hours)	0.44 ± 0.10
ASDAS-ESR	2.76 ± 0.60
ASDAS-CRP	2.78 ± 0.62
Bone merrow lesions	17.88 ± 10.63
Synovitis	8.80 ± 3.52
HIMRISS	26.67 ± 11.90
Treatment	
DMARDs (MTX + SSZ)	61 (31.3%)
Adalimumab	41 (21%)
Infliximab	8 (4.1%)
Etanercept	85 (43.6%)

### Annotation and definition procedure

The contours of the femur, femoral head, bone marrow edema, and synovitis were manually delineated on MRIs by two experienced readers (YZ and KZ) using Matlab software and RadiAnt DICOM Viewer (32-bit). Images were examined by three expert orthopedists and rheumatologists (QH, YL, and PZ). All disagreements were settled before training and testing.

### HIMRISS scores and clinical manifestation evaluation

MRIs were obtained with a coronal short-tau inversion recovery (STIR) sequence (STIR), scans with slice thickness of 4 mm, field of view of 400 mm × 400 mm, matrix size of 512 × 256 (STIR) or 512 × 307 (T1), repetition time of 3,550, echo time of 51, and inversion time of 145 ms, field of view 400 mm × 400 mm. The HIMRISS protocol was used to score the inflammatory changes in MRIs of the hip joints including BME of femoral head, BME of the acetabular, and synovitis (Zheng et al., 2019). The HIMRISS procedure was performed by two experienced readers (YZ and KZ) independently. The procedure was blinded to the treatment arm and MRI time point, and analyzed by an independent statistician. The standard HIMRISS scores of each patient were calculated based on five central MRI slices and an extra five front and five back slices. Due to the individual differentiation from real-word clinical operation, hip MRIs from different patients could not obtain enough slices. To avoid statistical bias, patients with MRIs of hip structure <5 slices were excluded as missing samples.

## Deep-learning network

### Data normalization

**FIGURE 1:**
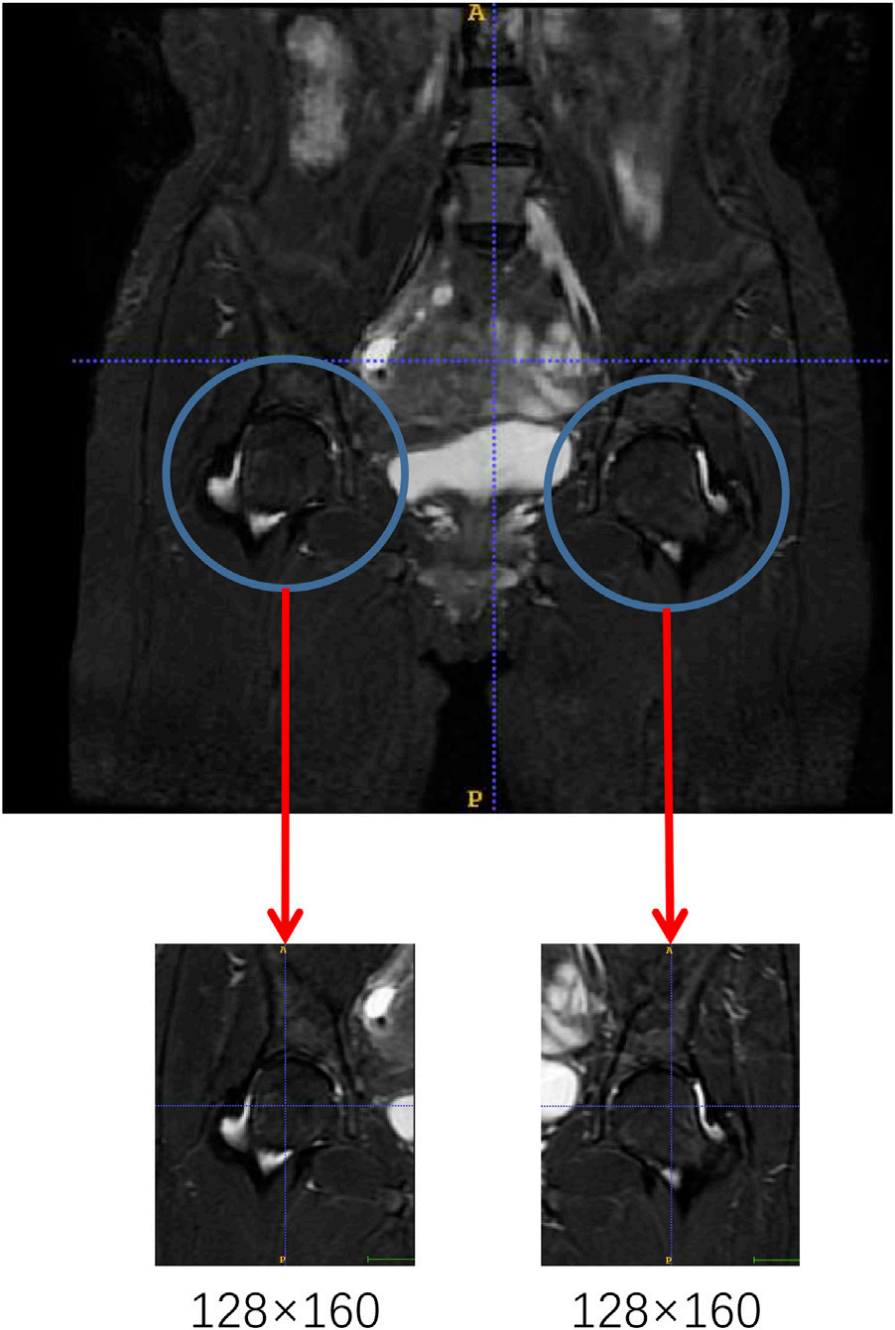
Image preprocessing procedure.

All of the raw images were preprocessed due to differences in width and height sizes. A specialized musculoskeletal radiologist excised the two femoral heads in the original images with 128 × 160 resolutions as network input data (Figure 1). This process ensured the same resolution of each image and minimized the noise interference caused by the bladder part in the model training.

### Network structure

**FIGURE 2:**
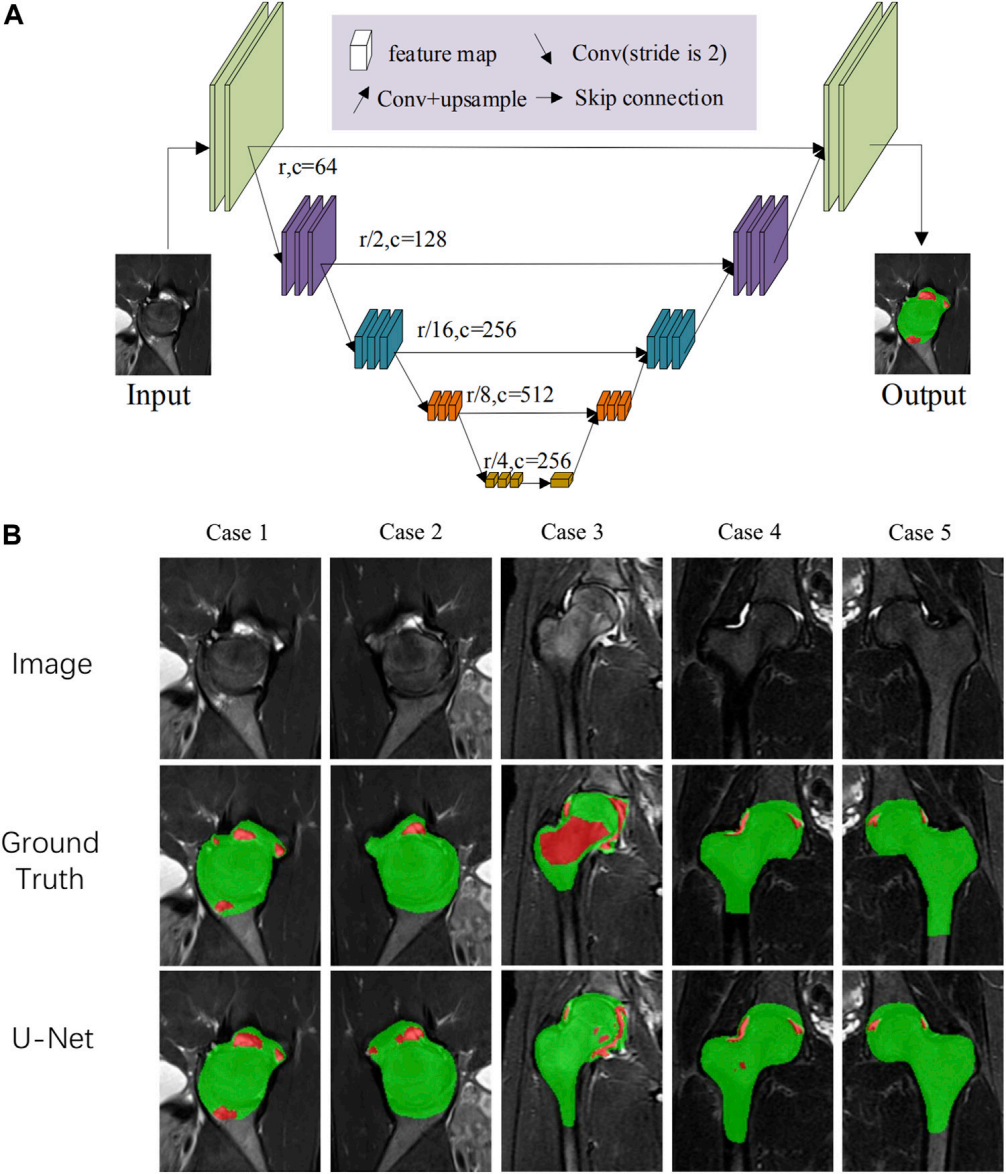
**(A)** The network architecture. U-Net was chosen as the backbone network for rough segmentation and input. The data were first imported to the U-Net network. After downsampling and upsampling, coarse prediction underwent a three-step point rendering, generating pixel-level segmentation. **(B)** The segmentation and visualized results of the U-Net network.

First, we trained multiple prediction networks to compare the segmentation accuracy, including U-Net, UNet++, Attention-UNet, and HRNet. The accuracies are summarized in Table 2. Results indicate that U-Net could achieve the best prediction accuracy. Therefore, U-Net was chosen as the backbone network to improve its performance. The network module of U-Net has an encoder-decoder structure, where the encoder extracts image features, and the decoder gradually restores the spatial resolution to generate segmentation probability maps. Moreover, the decoder stitches feature maps from the same stage encoder through skip connections to supplement the spatial information lost in the downsampling process. The encoder contains several downsampling modules, each consisting of two 3 × 3 convolutional layers with batch normalization, a rectified linear unit (ReLU) activation, and a 2 × 2 Maxpooling layer. The decoder contains some upsampling modules, each consisting of a transposed convolutional layer and two 3 × 3 convolutional layers with batch normalization and ReLU activation. At the end of the model, there is a segmentation head to generate pixel-level segmentation results (Figure 2A; Supplementary Table S1).

**TABLE 2:** Performance comparison of different backbone networks. Four different networks were assessed (UNet, UNet++, Attention-UNet, and HRNet).

Model	Femoral dice (%)	Lesion dice (%)
UNet	88.4839	69.3622
UNet++	87.8504	69.8737
HRNet	88.6196	69.1818
Attention_UNet	87.8258	69.1622

### Network training and validation

Images of individual femoral head after cutting were used as input data for the network. To reduce bias and increase model stability, the data were randomly divided into training and independent validation datasets in a five-fold cross-validation (Table 3).

**TABLE 3:** Five-fold cross-validation results of the U-Net backbone network.

Fold	Femoral dice (%)	Lesion dice (%)
Fold_1	88.4839	69.3622
Fold_2	89.6641	66.6253
Fold_3	88.7303	68.7982
Fold_4	87.2320	67.8388
Fold_5	88.6699	68.0801
Average	88.5560	67.5409

### Evaluation of clinical association

When the prediction was completed, the model generated the files presented with the prediction results and ground truth delineation in a visualized JPG format. Three experienced orthopedists and rheumatologists examined the prediction results and provided feedback on whether the model accurately identified BME and synovitis lesions, and whether it outlined the lesion correctly when compared with human delineation. The specification and sensitivity of evaluations from each expert were subsequently calculated.

### Statistical analysis

Statistical analysis was performed using GraphPad Prism version 8.0.0 for Windows (GraphPad Software, San Diego, CA, United States; www.graphpad.com). For continuous variables, such as the age of participants, an unpaired *t*-test was used to compare the difference between the training and validation groups, and presented as the mean ± standard deviation. For categorical data, such as the number of patients, chi-square tests were used to analyze the relationship between groups, and presented as the amount and proportions. The difference between the prediction results of different models and the ground truth were calculated and analyzed using a *t*-test. The significance level for all of the analyses was set at *p* < 0.05. Python version 3.6 was used to evaluate model prediction accuracy. Additionally, manually delineated MRI images were used as ground truth by Python version 3.6.

## Results

### Clinical characteristics and MRI annotations of the study population

A total of 195 patients were included in this retrospective study (male 79.2%, age 28.63 ± 12.04) and general characteristics of study participants are summarized in Table 1. Clinical symptoms analyzed included disease duration, hip function Harris score, disease activity of Bath Ankylosing Spondylitis Disease Activity Index (BASDAI), and Ankylosing Spondylitis Disease Activity Score (ASDAS). The laboratory indexes assessed included B27, erythrocyte sediment rate (ESR), and C-reactive protein (CRP). In addition, hip MRI inflammatory scores from 1945 slices, including HIMRISS, bone merrow lesion scores, and synovitis scores, were assessed by two experienced radiologists and agreed.

### Inflammation segmentation results of different models

MRI data input into the model refer to the MRI slice selection procedure of HIMRISS. We checked the accuracies of the segmentation results of femoral head and inflammatory lesions, including BME and synovitis from different networks (Table 2). Results indicated that the U-Net module had the best average segmentation accuracy of 88.48% for the femoral head and 69.36% for inflammatory lesions. Figure 2B shows some of the segmentation results of the U-Net network compared with manual annotation by radiologists. From case 1 and case 2, we found that BME in the femoral head and synovitis in the joint space or acetabulum could be segmented, especially in small lesions. The segmentation result of case 3 also showed that this model could segment the large area BME in the femoral head and femoral neck.

Based on these results, we chose U-Net as the backbone network to optimize its accuracy by five-fold cross-validation (Table 3). Cross-validation results indicated that U-Net could achieve a stable segmentation accuracy, with the accuracy of femoral head segmentation varying from 87.23% to 89.66%, while the accuracy of inflammation segmentation varied from 66.63% to 69.36%. Inflammatory lesions included BME in the femoral head and synovitis in the joint space. The irregular external profile of the joint and incomplete hip dissection results from multiple MRI slices may limit and lead to a relatively lower segmentation accuracy of inflammatory lesions.

### Systemic inflammation evaluation agrees with HIMRISS results

By integrating multiple hip MRI slices from a single patient, our model system could systematically and quantitatively assess hip lesions in patients. In order to systematically quantify inflammation in the BME and synovitis of the femoral head for a patient, five central slices as well as five slices from the anterior and five slices from the posterior were counted and evaluated based on HIMRISS procedures. Based on the HIMRISS image selection procedure, UNet-scores were calculated based on the same MRI slices by summing up the gray value proportion of the segmented lesion regions relative to the whole femoral head for each slice of every patient. Then, we evaluated the correlation between automatic network identification results with HIMRISS and clinical characteristics, including inflammatory indexes and disease activities (Table 4).

**TABLE 4:** Correlation between the U-Net quantification result and HIMRISS. Note, *p levels <0.05, **p levels <0.01, --P levels >0.05. Unetscore: Inflammatory score from U-net model; ESR, erythrocyte sediment rate; CRP, C-reactive protein; BASDAI, Bath Ankylosing Spondylitis Disease Activity Index; ASDAS, Ankylosing Spondylitis Disease Activity Score.

	UNet-score	BML	Synovitis	HIMRISS	CRP	ESR	Harris	BASDAI	ASDAS-CRP	ASDAS-ESR
**UNet-score**										
**BML**	**									
**Synovitis**	**	**								
**HIMRISS**	**	**	**							
**CRP**	*	--	--	--						
**ESR**	*	*	**	**	**					
**Harris**	--	**	*	**	**	**				
**BASDAI**	*	*	**	**	--	--	**			
**ASDAS-CRP**	--	*	**	**	**	**	**	**		
**ASDAS-ESR**	--	--	**	*	**	**	**	**	**	

Results indicated that our UNet-score significantly correlated with manually evaluated MRI scores, including the BME score (*p* < 0.05), synovitis score (*p* < 0.01), and HIMRISS score (*p* < 0.01). HIMRISS scores, including BME score and synovitis were also significantly with CRP, ESR and disease activity scores of BASDAI and ASDAS ((*p* < 0.01). The UNet-score showed significant correlation with BASDAI disease activity (*p* < 0.05). These results indicate that our model could achieve trustworthy clinical agreement and significant correlation with disease activity and HIMRISS scores. Our deep learning-based automatic hip inflammation evaluation model has the potential to relieve doctors’ burdens to assess SpA disease progression to some extent.

## Discussion

Hip inflammation is one of the most common complications in SpA, with more than half of SpA-hip patients having bilateral lesions, leading to a poor prognosis (Vander et al., 2013; Lopez-Medina et al., 2020). Hip involvement further limits quality of life and physical function associated with SpA itself, resulting in difficulties with walking, running, sitting, and sleeping (Soulard et al., 2021). The prognosis of SpA patients with hip involvement is worse than those with SpA alone, which needs to be emphasized in long-term clinical management (Lopez-Medina et al., 2020; Zhang et al., 2021). However, the degree of pain tolerance in SpA patients may significantly vary among individuals. Therefore, clinical symptom assessment combined with imaging evaluation, especially MRI, is crucial to accurately judge inflammation progression of the disease, and develop personalized treatment plans for SpA patients with hip involvement.

MRI allows for the early detection of hip involvement. Joint effusion/synovitis has been described as a frequent MRI finding with fluid surrounding the femoral head and femoral neck on T2WI or STIR images. In addition, subchondral BME is also a frequent finding characterized by hyperintense signals on STIR images and/or on contrast-enhanced T1-weighted fat-saturated images (Huang et al., 2013; Chen et al., 2016). In our previously published findings from an observational study in AS patients with early hip involvement, 42% of AS patients with minimal or no hip pain had one or more MRI lesions, while 56.7% of AS patients had no or mild hip pain (Han et al., 2021b). These results emphasized the sensitivity and importance of MRI in the early detection and diagnosis of hip involvement for SpA patients.

Many studies have been applied to the construction and application of hip MRI assessment methods, especially systematic quantification methods. HIMRISS is an important quantitative assessment of hip BME and synovitis based on global MRI images (Jaremko et al., 2016), and it has significant reliability with the BASRI-hip system and disease activity (Man et al., 2021). Our previous study demonstrated that HIMRISS could be used to assess hip inflammatory lesions in SpA-hip patients with significant intra-group consistency and clinical relevance (Lopez-Medina et al., 2020). However, HIMRISS was conducted based on manual annotation and image processing, which relies on the experience of clinicians. To further improve the efficiency and large-scale clinical application of HIMRISS, further optimization of its evaluation process is needed.

In recent years, deep learning algorithms for imaging segmentation have gained attention due to fast efficiency and stable accuracy to improve clinical practice (Lundervold and Lundervold, 2019). Work by von Schacky et al. developed a multi-task deep learning model for grading the severity of femoral osteophytes, acetabular osteophytes, joint-space narrowing, subchondral cysts, and subchondral sclerosis of hip osteoarthritis on radiographs (von Schacky et al., 2020). This study achieved a similar performance to expert radiologists; however, their multi-task system designed for hip joint lesions is not suitable for SpA patients, especially SpA patients with early coxitis. Our previously published work was the first to construct a full convolutional neural network model based on grand multi-scale convolution for segmentation of BME regions (Han et al., 2021a). This 3D-UNet model could capture sufficient scale information and help the segmentation of BME with different scales, shapes, intensity values, or random location distributions. However, further work needs be done to improve the quantitative results, assessment range, and computational accuracy of deep learning algorithms.

This study constructed an MRI quantification evaluation model of hip BME and effusion/synovitis based on the U-Net network. Through precise segmentation of inflammatory lesions and quantitative evaluation of the lesion volume, the U-Net model provides an important reference for the quantitative evaluation of early hip involvement in SpA. Figure 2A shows that the distribution of BME and synovitis in the hip joint was highly heterogeneous, and the external contour of the femoral head also changed significantly in different MRI slices. Our results suggest that, compared with other network modules, the U-Net network has the highest segmentation accuracy for inflammatory lesions and the external contour of the hip joint, and stable segmentation accuracy can still be obtained after 5-fold cross-validation.

Additionally, the UNet-score obtained through this automatic and quantitative evaluation model significantly correlated with the HIMRISS score (Table 4). This suggests that our machine learning method can achieve a similar evaluation performance to that of experienced radiologists, and it greatly improves the clinical diagnosis efficiency and need for physician dependence. Furthermore, the UNet-score significantly correlated with disease activity based on clinical symptoms, suggesting that the model was clinically interpretable and could provide an important reference for precise clinical evaluation of hip inflammation in SpA.

Despite these novel findings, this work has some limitations. First, in addition to the most common bone BME and effusion/synovitis, the early manifestations of hip inflammation can accompany by fat deposition, a thickened synovium, bone erosion, bone proliferation, muscle involvement, enthesitis, bony ankylosis, and other symptoms. In future work, we will continue to develop a multi-task assessment model to more comprehensively diagnose and assess MRI manifestations of hip inflammation. In addition, validation in a larger study with more samples is needed to evaluate and improve the stability, accuracy, and validity of the model.

## Conclusion

Our work demonstrates the feasibility of an automatic deep learning method to systematically quantify BME and synovitis of hip inflammation, and it showed a significant similar performance to that of expert radiologists. This work could provide an efficient and reliable imaging evidence system to assess changes in hip inflammation during long-term follow-up, and the formulation of personalized treatment plans for SpA patients.

## Data Availability

The raw data supporting the conclusions of this article will be made available by the authors, without undue reservation.

## Abbreviations

SpA, Spondyloarthritis; MRI, Magnetic Resonance Imaging; ESR, Erythrocyte Sediment Rate; CRP, C-Reactive Protein; HIMRISS, Hip MRI Inflammation Scoring System; ASAS, Assessment of Spondyloarthritis international Society; STIR, Short-Tau Inversion Recovery; T2WI, T2 weighted image; ReLU, Rectified Linear Unit; BASDAI, Bath Ankylosing Spondylitis Disease Activity Index; ASDAS, Ankylosing Spondylitis Disease Activity Score; UNet-score, Inflammatory score from U-net model.
