# Supplementary material for: Deep-learning based quantification model for hip bone marrow edema and synovitis in patients with spondyloarthritis based on magnetic resonance images
Source: Front Physiol. 2023 Mar 3;14:1132214. doi: 10.3389/fphys.2023.1132214 (PMC10020192; doi:10.3389/fphys.2023.1132214)
Supplement: Supplementary file 1 [file Table1.DOCX]

**Table S1:The detailed parameters of UNet.**

|  | **Input size** | **Output size** | **Input channels** | **Output channels** | **Normalization** | **Kernel size** |
| --- | --- | --- | --- | --- | --- | --- |
| **Conv0_0** | **128×160** | **128×160** | **1** | **32** | **BatchNorm2d** | **3×3** |
| **Conv1_0** | **128×160** | **64×80** | **32** | **64** | **BatchNorm2d** | **3×3** |
| **Conv2_0** | **64×80** | **32×40** | **64** | **128** | **BatchNorm2d** | **3×3** |
| **Conv3_0** | **32×40** | **16×20** | **128** | **256** | **BatchNorm2d** | **3×3** |
| **Conv4_0** | **16×20** | **8×10** | **256** | **512** | **BatchNorm2d** | **3×3** |
| **Conv3_1** | **16×20** | **16×20** | **768** | **256** | **BatchNorm2d** | **3×3** |
| **Conv2_2** | **32×40** | **32×40** | **384** | **128** | **BatchNorm2d** | **3×3** |
| **Conv1_3** | **64×80** | **64×80** | **192** | **64** | **BatchNorm2d** | **3×3** |
| **Conv0_4** | **128×160** | **128×160** | **96** | **32** | **BatchNorm2d** | **3×3** |
| **Final** | **128×160** | **128×160** | **32** | **3** | **/** | **1×1** |
